# Do Impressions on the Mother Affect the Unborn Child

**Published:** 1886-03

**Authors:** 


### DO IMPRESSIONS UPON THE MOTHER AFFECT THE UNBORN CHILD.

C. W. Baker, M. D., makes the following statement in regard to this mooted question among medical men, and very truly remarks that in every neighborhood you can hear stories that sound sensational.

"In this neighborhood a very respectable lady tells of her sister stepping on a coon hide that the children had sloughed, and she gave birth to a child that looked like a coon."

"I have just had a case that strengthens my hitherto weak faith in such impressions affecting the unborn. On the night of November 27th, I was called to attend Mrs. B., age 30, the mother of four strong, healthy children ; in half an hour after I arrived at the house I succeeded in delivering her of a female child, weighing eleven pounds, that had a head that very much resembled a snake's head; all of the head above the eyes and ears was wanting, also the back of the head; what little brain there was, was not covered with cranium on top or at the back part."

"Its motions resembled those of a serpent; whenever it was touched it would squirm and dart out its head so it was impossible to feed it; then the noise it made resembled the hissing of a snake more than the crying of a baby, and was so shocking to those that heard it, that they kept it covered as tight as possible in bed. It died in forty-eight hours."

Mrs. B. was called sometime in May last by a neighbor woman to help kill a snake; it was over six feet long and so vicious that the women had to call the men to help them. Mrs. B. was nearly prostrated with fright.

"The practical part of this is, that women should keep away from scenes that are not pleasant, when they are pregnant; and can they not have some influence in shaping the destiny of the child, by cultivating such thoughts and actions while pregnant, as they would admire in the child?"

"It is a fact of history that while Napoleon was in the embrionic state, his mother took her dead husband's place at the cannon in defending their little island. Is it strange he was such a warrior?

